# Navigating artificial intelligence in home healthcare: challenges and opportunities in nursing wound care

**DOI:** 10.1186/s12912-025-03348-7

**Published:** 2025-06-19

**Authors:** Sara Karnehed, Ingrid Larsson, Lena Petersson, Lena-Karin Erlandsson, Daniel Tyskbo

**Affiliations:** School of Health & Welfare, Halmstad University, Halmstad, Sweden

**Keywords:** Artificial intelligence, Machine learning, Digitalization, Home healthcare, Municipal care, Wound care, Nursing, Nursing practice

## Abstract

**Background:**

Artificial intelligence (AI) is increasingly introduced into healthcare, promising improved efficiency and clinical decision-making. While research has mainly focused on AI in hospital settings and physician perspectives, less is known about how AI may challenge the values that guide nursing practices. This study explores nurses’ perceptions of wound care in municipal home healthcare and the opportunities and challenges with the integration of AI technologies into their practices.

**Methods:**

An exploratory qualitative study using semi-structured interviews was conducted with 14 registered nurses from two municipalities in Sweden. Participants were recruited through purposive sampling, and data were collected through individual interviews, either in person or via video call. Interviews were transcribed verbatim and analyzed inductively, inspired by the Gioia methodology. This approach allowed themes to emerge from the data while maintaining close alignment with participants’ perspectives. In a subsequent phase, the data were interpreted through the lens of Mol’s *Logic of Care* to deepen understanding of the relational, embodied, and adaptive nature of wound care. Ethical approval was obtained, and the study adhered to the Consolidated Criteria for Reporting Qualitative Research (COREQ).

**Results:**

Three interconnected dimensions emerged from the data: relational, embodied, and adaptive practices. Nurses emphasized the importance of relational work in wound care, highlighting the trust and continuity necessary for effective wound care, which AI-driven automation might overlook. Embodied practices, such as sensory engagement through touch, sight, and smell, were central to wound care, raising nurses’ concerns about AI’s ability to replicate these nuanced judgments. Adaptive practices, including improvisation and situational awareness in non-standardized home environments, were presented as challenges for AI integration, as existing digital systems were perceived as rigid and often increased administrative burdens rather than streamlining care.

**Conclusions:**

Home healthcare nurses’ perspectives highlight the complex interplay between technology and caregiving. While AI could support documentation and diagnostic processes, its current limitations in relational, sensory, and adaptive aspects raised the nurses’ concerns about its suitability for wound care in home settings. Successful AI integration should account for the realities of nursing practice, ensuring that technological tools enhance the embodied, relational, and adaptive dimensions of wound care. Applying Mol’s Logic of Care helps illuminate how good care emerges through ongoing, situated practices that resist full automation. Future research could further explore how AI aligns with professional nursing values and decision-making in real-world care settings.

**Clinical trial number:**

Not applicable.

**Supplementary Information:**

The online version contains supplementary material available at 10.1186/s12912-025-03348-7.

## Introduction

The rapid development and introduction of advanced digital technologies, such as artificial intelligence (AI), in healthcare have generated substantial expectations regarding their potential to revolutionize healthcare delivery [1, 2]. These technologies, with capacity for deep data analysis, offer promising avenues for enhancing nursing practices by supporting more informed decision-making and optimizing treatment processes [3–5]. However, the integration of AI into nursing practice also presents significant challenges [6, 7]. Notably, many AI technologies are implemented without involving nurses in their design and development [8, 9], which raises concerns about their compatibility with nursing values and practices [10, 11].

While much of the existing research has primarily focused on physicians’ perspectives, particularly in hospital settings [12–14], there is a considerable gap in understanding the perspectives of nurses and the implications of AI technologies in home healthcare practices [15, 16]. This omission is striking, given that nurses in home healthcare often work in complex care contexts, i.e. in private homes not equipped for advanced medical treatment, combining technical expertise and relational engagement.

In Sweden, as in many other countries, home healthcare nurses provide critical care to patients in their homes. One frequent task for home healthcare nurses is the treatment of hard-to-heal wounds, including malignant wounds, pressure ulcers, arterial and venous ulcers, and diabetic foot ulcers [17, 18]. Research has shown that nurses in home healthcare often have insufficient knowledge of wound care, and the topic receives little emphasis in nursing education [19–22]. This lack of knowledge contributes to poor wound healing, frustration among healthcare professionals, and prolonged patient suffering [23, 24]. Efforts to introduce AI technologies in wound care have recently gained significant traction [25–28]. Such technologies aim to enhance clinical outcomes and reflect broader professionalization initiatives within wound care, where technological advancements are positioned as markers of expertise and innovation [29].

AI technologies are not value-neutral; rather, they function as sociotechnical systems composed of diverse social, political, economic, cultural, and technological factors that inherently carry embedded norms, values, and logics, such as managerialism or technical priorities [30–33]. Consequently, these systems may challenge the values that guide nursing practices. In addition, previous research demonstrates that technologies often embody institutional logics that can disrupt established professional norms and practices [34–36]. As healthcare is a human-centered practice based on interrelations and tactile understanding, where judgment, intuition, and common sense are pivotal, the automation of work is regarded as particularly complex to achieve [37, 38]. Recent research by Kuijper et al. [39] further stresses the relational aspects of caregiving, demonstrating how care practices are co-constructed through collaboration between caregivers, patients, close family, and non-human actors like technologies. In nursing, this raises important questions about how the values embedded in AI technologies conform to the practices of caregiving, particularly in home healthcare settings where context-specific care is central. Considering the growing interest in AI-driven wound care, it is essential to explore home healthcare nurses’ perceptions of wound care and the potential opportunities and challenges associated with AI integration.

In Sweden, the implementation of AI in home healthcare is still in its early stages, with most technologies limited to pilot or development phases [40]. Current initiatives primarily involve AI-based image recognition for wound assessment, automated medication dispensers, and digital decision support systems. However, these tools are rarely integrated into routine municipal care. Instead, municipalities often rely on basic digital systems for documentation and communication, some of which are labeled as AI despite lacking advanced machine learning capabilities. Notable examples include a Vinnova-funded pilot using AI to classify ulcer types from wound images [41], and ongoing European projects exploring AI-supported rehabilitation using motion tracking [42] wearable sensors for monitoring anxiety in older cancer survivors [43], and real-world data–based decision tools for managing chronic conditions in home care [44]. While these projects highlight the potential of AI, large-scale adoption remains limited, revealing a persistent gap between technological promise and everyday practice. Thus, this study aimed to explore home healthcare nurses’ perceptions of wound care and the opportunities and challenges posed by AI integration into wound care practices.

## Methods

### Research design

An exploratory qualitative research design was applied, inspired by the Gioia methodology for inductive theory development [45]. This approach enabled a structured, multi-stage analysis that moved from participants’ own terms and experiences toward more abstract conceptual themes. To ensure methodological rigor, the study is reported following the Consolidated Criteria for Reporting Qualitative Research (COREQ) checklist [46] (see additional file 1).

### Setting

This qualitative study was conducted in the home healthcare organizations of two municipalities situated on the Swedish West Coast. Each municipality covers approximately 1,000 square kilometers with a population density of 11 and 28 inhabitants per square kilometer, respectively. Registered nurses (referred to hereafter as “nurses”) in these settings were responsible for patients’ healthcare. Home healthcare differs from hospital care in that personnel, materials, and diagnostic instruments must be transported. The conditions also differ spatially, as often home environments are not optimally designed for healthcare, lacking features such as hygienic equipment and ergonomically supportive settings.

### Sample

A purposeful sampling approach was employed to recruit nurses from the two municipalities. Participants were recruited based on the following inclusion criteria: [1] registered nurses working in the municipality’s home healthcare organization, and [2] experience in wound care within home healthcare settings. No additional formal exclusion criteria were applied beyond these conditions. The researchers contacted the managers of each home healthcare organization and asked for assistance in recruiting nurses. Participants were mailed an invitation and information sheet. Nurses who declined to participate attributed their decision to competing commitments, such as work obligations. The final sample consisted of 14 nurses; 13 women and one man. The average age of the participants was 43 years, ranging from 24 to 59 years. Nine of the nurses held a bachelor’s degree in nursing, and five had a master’s degree in nursing. One participant had completed a dedicated university-level wound care course, and all participants had received some theoretical wound care training as part of their nursing education. The participants had individual experience in wound care ranging from one to 27 years. They engaged in wound care activities one to five days per week. This ensured a diverse range of insights into wound care practices.

### Data collection

Individual interviews were used as a data collection method, aiming to elicit detailed insights into participants’ descriptions of wound care and their perceptions of the integration of AI technologies. Individual interviews were conducted to enable confidential and in-depth reflection on professional experiences, including potentially sensitive perspectives on organizational conditions, clinical judgment, and technology use. The interviews were held in Swedish by an independent, experienced researcher (DT) (*n* = 3) and by a supervised researcher experienced in home healthcare and interviewing (SK) (*n* = 11). A total of 14 interviews were completed, with sessions held either on-site at the workplaces of nurses (*n* = 5) or via video call where necessary due to logistical considerations (*n* = 9). No one else was present during the interviews other than the participant and researcher, and no prior relationships existed between the researchers and participants. The interviews took place between spring and fall of 2023. The interviews were conducted using an interview guide consisting of semi-structured, open-ended questions addressing perceptions of wound care practices and AI (see Additional File 2). The interview guide was developed specifically to align with the study objectives, enabling participants to openly describe their experiences and perspectives regarding wound care and technology without being constrained by predefined theoretical categories. This open-ended approach is consistent with the Gioia methodology, which emphasizes capturing participants’ lived experiences while allowing flexibility for emergent themes [45]. The interview guide was pilot tested in two interviews, which confirmed that the questions effectively elicited relevant responses and required no further adjustments. Follow-up questions were employed during interviews to explore or clarify points further when necessary. All interviews were audio-recorded, with each session lasting approximately one hour.

### Data analysis

The interviews were transcribed verbatim and manually by a trained research assistant, and subsequently analyzed by the researchers who were inspired by the method outlined by Gioia [45, 47, 48]. This method allows for an inductive process where themes emerge from the data, ensuring that the theoretical insights are firmly rooted in the participants’ perceptions, while also contributing to broader theoretical understanding.

The initial stage of analysis involved focusing on the transcribed interviews. At this stage, the researchers stayed close to the expressions of the informants, treating their narratives and terminology as key indicators of meaningful themes. The transcripts were managed and coded using Microsoft Word, which supported systematic tracking of first-order concepts and emerging patterns. After that, the researchers moved to a more interpretive and iterative stage of the analysis, making sense of the data by developing second-order themes representing deeper structures or patterns in the data. In the later stages, this process became increasingly iterative, with continuous movement between data and theory to refine our conceptual understanding and develop aggregate dimensions. This allowed us to recognize the value of relating our insights to Mol’s analytical approach to the logic of care [49], which emphasizes how good care is enacted through ongoing adjustments, relationships, and situational responses. The approach provided a theoretical lens for understanding how wound care was practiced. Through this inductive process, we identified core themes within the data, which were later examined through the lens of Mol’s concept of the logic of care. This allowed us to situate our findings within broader theoretical discussions about care practices and the integration of technology in nursing.

**Fig. 1:**
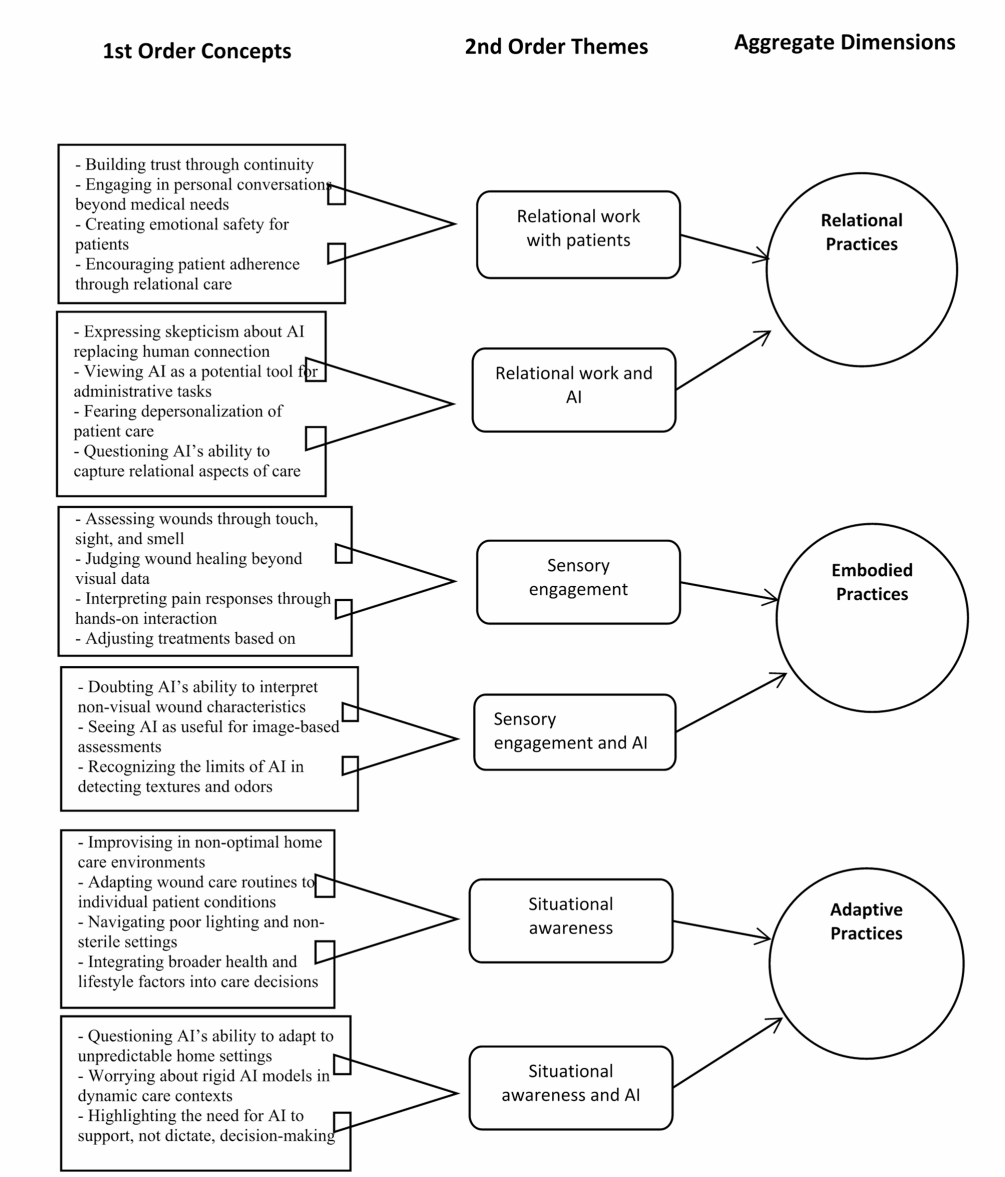
Data structure illustrating the progression from raw data to aggregate dimensions, showing the relationship between first-order concepts, second-order themes, and aggregate dimensions

During analysis, agreements were reached through data revisits and discussions between all authors, who have extensive experience in research regarding AI implementation in healthcare and qualitative methodology. Data collection and analysis occurred iteratively. We concluded data collection when no new first-order concepts were emerging and the development of second-order themes and aggregate dimensions stabilized. Coding and theory development were considered complete once we were satisfied with the thematic clarity and the data structure had reached theoretical saturation—that is, when no new insights or relationships emerged, and the structure was conceptually robust and internally coherent [45]. This judgment was also consistent with broader qualitative guidance on assessing thematic saturation [50]. The progression from raw data to themes and dimensions is represented in Fig. 1, which not only visualizes the relationship between second-order themes and aggregate dimensions but also serves as the structural foundation for the results section.

### Ethical approval

To ensure the study adheres to ethical principles, it has been conducted in accordance with the ethical guidelines outlined in the Declaration of Helsinki, encompassing information, consent, confidentiality, and benefit [51]. Approval was obtained from the Swedish ethical review authority (Approval No. 2022-05837-01). Both oral and written informed consent were obtained from all respondents prior to participation, ensuring they were fully aware of the study’s purpose, procedures, potential risks, and their rights to withdraw at any time. Data were stored according to data protection regulations.

## Results

This section presents findings organized into three interconnected aggregated dimensions: Relational practices, Embodied practices, and Adaptive practices. These dimensions reflect how nurses perceive wound care and the opportunities and challenges posed by AI integration into those practices. Each aggregated dimension consists of two second-order themes. The first second-order theme within each dimension focuses on how nurses describe their wound care practices, emphasizing relational, sensory, or situational aspects of their work. The second second-order theme examines how nurses perceive AI in relation to these specific practices, reflecting on its potential to support wound care practices. The results are illustrated with quotes from participants, referred to as P1, P2, etc. in the order they are included in the paper.

### Relational practices

Relational care emerged as a central theme, reflecting the importance of trust-based, person-centered approaches in wound care. Nurses emphasized the relational work with patients and their care teams which they viewed as crucial for clinical outcomes.

While AI could assist with administrative or diagnostic tasks, nurses strongly believed that relational aspects of care could neither be fully replicated by technology, nor should these be compromised in the pursuit of technological efficiency.

#### Relational work with patients

Nurses described relational work with patients as foundational in wound care, achieved by fostering continuity and ensuring that the same nurse made home visits to each patient. Relationships built on trust, familiarity, and empathy were considered essential not only for emotional support but also for improving clinical outcomes. Nurses described how wound therapies could be uncomfortable and painful for patients. Acknowledging this suffering and building relationships was essential to make the patients trust the nurses and align with the treatment. The lack of a relationship, by contrast, often resulted in patient discomfort and mistrust. One nurse emphasized the importance of relations:

*Yes*,* if it’s the same person who comes*,* they don’t have to explain every time that it should be done in this or that way. But you know that you are showering*,* you take your pain relief*,* we do it on the chair or you lie in bed*,* you put anesthesia first*,* you wait. It becomes routine. It becomes security. And you have this little moment to talk to each other as well. These are the kinds of things that are important*,* even though they are not so visible.* P1.

Relational work also extended to understanding patients’ social contexts and everyday realities through personal conversations that went beyond immediate medical needs. These interactions allowed nurses to provide tailored care that adapted to patients’ unique circumstances, fostering a sense of safety and collaboration.

*This may not sound very professional*,* but it’s not just a care relationship*,* it’s also like ‘here comes a good friend*,* oh*,* how nice it is to chat for a while’. I think that part is important. That you have a mutual feeling for each other in some way and build relationships.* P2.

The relational dimension of nursing also extended to collaboration within the care team, emphasizing the collective nature of care. Nurses described how they worked closely with nurse assistants, social workers, and physicians to ensure continuity and coherence in patient care. They discussed patient needs and strived for transparent and clear communication. The nurses described themselves as being at the center of the care team, advocating for patients and delivering information from different caregivers to others.

#### Relational work and AI

Some nurses recognized the potential of AI systems to assist with specific administrative and diagnostic tasks, such as tracking wound healing, supporting diagnosis, and streamlining documentation. These tasks, they noted, were less reliant on human relationships and could theoretically free up time for more meaningful patient interactions. However, nurses consistently expressed concerns that AI technologies could depersonalize and dehumanize care by reducing patients to medical objects or data points. They doubted AI’s ability to provide the emotional and social support that they described as integral to their work and emphasized that technological tools should remain secondary to human engagement. As one nurse observed:

*I don’t know how to capture the human behavior in algorithms. I usually say*,* “Everyone gets old*,* both the odd ones and the non-odd”. We have all kinds of people here. I don’t think we can ever replace human contact and human closeness; our patients have a need for it*. P3.

Nurses also noted that current digital systems had not effectively reduced their workload or resulted in more time spent with patients, and they feared that AI might further exacerbate administrative burdens rather than alleviate them.

*I would probably say that it is between 75 and 50% of our time that we spend in our systems and doing documentation. That’s a lot of time*,* and I would have preferred to have more time with the patients*. P4.

Regarding the relational work within the care team, the opportunities for AI to facilitate information transmission between different caregivers to reduce gaps in patient data were highlighted. The nurses hoped for systems that could facilitate information exchange between different caregivers, which, for example, could give them access to wound experts or opportunities to be informed about patients’ daily status from the home care aids. However, these opportunities were regarded as difficult to achieve as patient security regulations at this point hindered different stakeholders, even within the same municipality, from accessing each other’s digital documentation. Thus, critical information beyond medical issues was retrieved by physical meetings or phone calls.

### Embodied practices

Wound care was described as physical work. Home healthcare nurses used their bodies to provide wound treatment and relied on their senses to judge wounds and patient status. They viewed those aspects of their work as difficult for AI to replicate.

#### Sensory engagement

When nurses were at a patient’s home to perform wound care, they inspected the wound both visually and physically to judge the state of the wound and the efficiency of the eventual treatment. Wound management was described as physical work that required them to engage in body work such as cleansing, removal of dead tissue, and dressing of the wounds. The nurses described how they relied on their senses, such as touch, smell, and sight, to gather critical information about a wound. They used their hands to feel the texture, firmness, and structures of the skin. This sensory involvement allowed for deeper insights beyond visual observation. For example, one nurse emphasized the importance of human touch:

*Yes*,* as I said*,* how does the skin feel? Is it rock-hard? Are the wound edges extremely hard*,* requiring loosening and removal? It’s very important to have a feel for it*,* and similarly*,* when you feel ‘this seems almost undermined underneath’*,* you might sometimes feel that ‘it sinks in when I press here*,* even though it looks completely fine on the surface’. So*,* I think it’s important to both smell and use this sense too.* P5.

#### Sensory engagement and AI

Nurses thought that future AI technologies could assist wound care by processing visual data but were unlikely to replace the multi-sensory embodied practice they performed. For instance, one nurse expressed skepticism about AI technologies’ ability to capture the complexity of wounds, particularly sensory details like smell or texture:

*A picture might make fibrin look yellow*,* but in fact*,* it’s gray-green. Or if I suspect pseudomonas*,* I can sometimes tell by the slightly unpleasant smell and the way the fibrin shimmers green in the wound.* P6.

The nurses envisioned a future where AI and other technologies acted as decision-support tools to enhance the sensory and embodied practices they regarded as central to wound care, rather than as replacements for their critical thinking and judgment.

*In some way*,* you would like to be able to get a suggestion for action. Or a suggestion on what kind of wound you have in front of you. It would have been ideal. Yes*,* like ‘this wound looks like it could be a venous ulcer’ for example. But it is an enormous job to get it done digitally*,* considering that every single wound is unique*. P7.

### Adaptive practices

Nurses frequently adapted their practices to respond to the unpredictable nature of home healthcare. Improvisation and real-time problem-solving were essential in navigating environmental challenges, patient needs, and resource constraints. This adaptability was seen as a crucial aspect of wound care, yet it also highlighted tensions with AI technologies, which often require standardized conditions to function effectively. Nurses expressed concerns about whether AI could accommodate the flexibility and situational awareness necessary for wound care in diverse home settings.

#### Situational awareness

Home healthcare settings posed significant environmental challenges, requiring nurses to adapt creatively to conditions in patients’ homes. Poor lighting, unsanitary spaces, and non-adjustable furniture were common obstacles, but nurses described how they found ways to work around these limitations.

*You may have nowhere to put your bag or set up your stuff in a home environment. Sometimes it’s a bad working environment and bad lighting*,* and it’s hard to get through. Yes*,* so it’s a big challenge that you don’t have a sterile table*,* for example*,* a stainless-steel table that you set up your stuff on or have the right lighting. So that’s probably the hardest part. You might pick up a kitchen chair to use as a table*,* so you simply have to improvise.* P8.

In this way, nurses responded flexibly to environmental constraints, ensuring that care remained person-centered even in challenging conditions.

Situational awareness also extended to a holistic understanding of patients, where nurses considered factors such as mental health, activity levels, and social context alongside physical symptoms. This broader perspective allowed nurses to address the root causes of wounds, rather than focusing narrowly on clinical interventions.

*It’s this thing about diet*,* it’s this thing about daily rest*,* night fasting*,* skin care*,* observation; that it is actually important. Because the best treatment for wounds is to take actions before it becomes a wound.* P7.

A holistic approach was described by the nurses as crucial for preventing wounds from occurring. However, they described that the organizational structure of care delivery limited their ability to work preventively. Some nurses described how they were told to focus on health problems that had already occurred and felt that this prevented them from forestalling wound assurance, leading to a conflict between professional judgment and organizational control.

#### Situational awareness and AI

The non-standardized nature of home healthcare posed challenges not only for existing digital tools but also for the prospective integration of AI technologies, which, in the nurses’ experience required a level of environmental and procedural standardization that may not be feasible in diverse, unpredictable care settings. Technologies in use, such as medical robot dispensers and electronic medication administration applications (eMAR), were described as task-oriented, targeting one specific problem, which often created a need for workarounds to make them suitable in their clinical practice. While discussing the potential role of AI technologies in wound care, nurses emphasized the significant variability among patients, and the complexity of the patients’ individual situations. This complexity often required holistic, adaptive approaches that went beyond the capabilities of single-purpose technologies.

*You have to remember that all people are different. Two patients with the same health problem may not be looking for the same solution. They may not have the same goals at all. And I think it can be difficult to relate to that with AI. If you take heart failure patients as an example*,* with leg swelling and all the problems that entails*,* some want to make all efforts and their goal is to get rid of the problems*,* while others just want to live with it and don’t want support stockings*,* don’t want diuretics and so on. It’s so different even though it’s the same problem. So*,* an AI must*,* in such cases*,* have the ability to take into account the patient’s own goals and the patient’s will. That’s what we’re doing today*,* we’re listening.* P3.

Nurses highlighted the critical role of human caregivers in adapting to patients’ unique needs and preferences, which stresses the challenges of implementing standardized technologies in diverse and nuanced care contexts. One nurse illustrated this concern with an example of the use of automated medication dispensers, noting the diverse patient responses:

*For those who are still using it*,* it has worked quite well. But some have sent them right back*,* and others have thrown them down the stairs.* P2.

#### Summary of findings

Together, the three aggregate dimensions of relational, embodied, and adaptive practices, capture how nurses perceive wound care and how they interpret the opportunities and challenges of AI integration into these practices. Across all themes, nurses stressed that good wound care involves trust-based relationships, hands-on sensory engagement, and responsiveness to patients’ individual situations. Opportunities for AI were mostly seen in supporting documentation, diagnostics, or communication, whereas concerns centered on the risk of losing flexibility, context-sensitivity, and human connection. While analytically presented as distinct, these dimensions are deeply interconnected in practice. For instance, embodied interactions (such as touch and smell) were described as vital for building trust, and situational adaptation was often tied to both relational knowledge and sensory assessment. This interplay reinforces the view that AI integration must account for the complex, interwoven character of professional nursing work, rather than treating these dimensions in isolation.

Wound care emerged as a continuous sequence of situated temporal responses where each dimension shaped and enabled the others. Nurses moved back and forth between relational, embodied, and adaptive dimensions, for example, returning to relational engagement when sensing patient discomfort, and revising embodied assessments due to patient feedback. This temporal layering reflects a choreography of care rather than a checklist of competencies.

## Discussion

This study explored home healthcare nurses’ perceptions of wound care and the opportunities and challenges posed by AI integration into wound care practices. The discussion is structured around the three core dimensions identified in the findings—relational, embodied, and adaptive practices. Each section begins by illustrating how nurses perceive their current wound care practices and then explores the opportunities and challenges they associate with integrating AI into those practices. This structure reflects the full scope of the study’s aim while maintaining thematic coherence.

Wound care in home healthcare, as described by the nurses, is fundamentally shaped by relational, embodied, and adaptive practices. Nurses emphasized the importance of relationships with patients, sensory engagement in wound assessment, and the need for continuous adaptation to unpredictable care situations. This shows that the process of wound care in home settings extends beyond the purely mechanical task of treating a physical wound. Instead, it encompasses a wide range of physiological, logistical, social, and emotional elements, highlighting that wound care does not merely represent a clinical procedure but serves as an interaction potential for the nurse and the patient.

Our findings emerged inductively from the nurses’ experiences and perspectives, and Mol’s work on the logic of care was applied as a theoretical lens to help frame and interpret these insights, offering a richer understanding of the relational, embodied, and adaptive dimensions in wound care practices. Rather than defining good care as a fixed standard, Mol emphasizes that good care is a situated, ongoing process that requires continuous adaptation, negotiation, and experimentation in response to uncertainty and competing priorities [44]. This perspective highlights how nurses’ professional judgment, patient relationships, and the specificities of home healthcare settings shape wound care in ways that cannot be fully standardized [44, 46]. Rather than imposing external values and pre-defined answers to what is right or wrong onto these practices, the approach seeks to make visible the values and considerations that healthcare professionals engage with in practice [46–49]. By applying this analytical lens, this study moves beyond simplistic evaluations of care quality. Instead, it reveals how nurses make sense of and negotiate their caregiving roles in response to technological change. This is particularly relevant to home healthcare, where nurses often operate in unpredictable conditions and must make context-specific decisions. Understanding how the complexity of care practice interacts with the structured logic of technology is essential to assessing AI’s role in nursing, particularly in relation to professional skills [50].

### Relational work in nursing: opportunities and challenges for AI

Nurses in this study described relational work as essential for building patient trust, improving treatment adherence, and achieving better outcomes in wound care. This aligns with previous research that emphasizes trust, empathy, and continuity as critical components of effective care [52, 53]. A recurring theme in the literature suggests that AI could automate routine and administrative tasks, freeing up time for nurses to engage in relational care [54–56]. Similar expectations were expressed by nurses in this study, who envisioned AI assisting with wound tracking, diagnostic support, and documentation, theoretically freeing up time for patient interaction. Importantly, our findings also nuance this optimistic narrative. Nurses reported that existing digital systems had increased their administrative workload rather than lowering it, limiting the time available for direct and relational patient care. This reflects broader concerns in healthcare technology research, where technological innovations often fall short in delivering the promised efficiencies and instead introduce new complexities and bureaucratic demands, ultimately reducing the time nurses can spend with patients, and increasing administrative tasks [57–59].

In our study, nurses emphasized the importance of maintaining relational aspects of care, expressing concerns that AI-driven tools risk prioritizing efficiency and standardization over the nuanced, context-sensitive interactions fundamental to wound care. Existing AI technologies can analyze vast amounts of textual, visual, and other patient data to deliver personalized information and recommendations [60]. Such capabilities could facilitate person-centered care by improving access to individualized health information and supporting informed decision-making, but only if relational aspects of nursing are actively incorporated into AI development [61]. To achieve this, it is essential for nurses to articulate how relational, caring-based approaches contribute to clinical outcomes in wound healing and potentially how these empathetic elements, often referred to as artificial empathy, can be integrated into AI-driven healthcare models. For example, research has shown that care robots can be programmed to display expressions that simulate empathy, making their interactions with patients feel natural and supportive [62]. However, nurse-led innovations often struggle to gain legitimacy within healthcare systems dominated by hierarchical knowledge structures that prioritize medical and technological expertise over nursing insights [39, 63]. This power imbalance, evident in the marginalization of nurses’ perspectives on AI implementation, limits their influence as change agents and stifles opportunities for organizational learning that could foster more holistic, patient-centered care models. Moreover, the ethical aspect of replacing human relationships with artificial empathy is not an obvious goal. Our findings show that nurses were concerned that AI technologies could depersonalize and dehumanize care by reducing patients to medical objects or data points. This devaluation of human roles in care has also been discussed in research. For example, Schultz et al. define the concept of algorithmic dehumanization as “the intentional or unintentional treatment of individuals and/or groups as less than fully human, thereby violating human rights, including liberty, equality and dignity” [64].

### Embodied practices in nursing: opportunities and challenges for AI

Our findings highlight that embodied practices, particularly sensory engagement through touch, sight, and smell, are fundamental to wound care in municipal home healthcare. Wound assessment is not purely visual. Nurses rely on these sensory interactions to assess wounds, detect subtle changes, and adjust treatment strategies based on real-time, hands-on evaluations. This aligns with previous research emphasizing that nursing work is deeply embodied, requiring professionals to use their senses and intuition to make clinical decisions in dynamic care environments [65–68]. In contrast, AI technologies are inherently incorporeal, lacking the embodied and relational capabilities fundamental to nursing care [69–71]. From this perspective, AI presents both opportunities and challenges. AI-based wound assessment technologies, often developed using image-based machine learning models, can enhance objectivity and support clinical decision-making [72, 73]. Moreover, machine learning algorithms using quantitative measures of epidermal moisture, which are hidden from the unaided eye, have been developed for early detection of pressure ulcers [74]. Emerging technologies, such as smart wound dressings and biosensors, are capable of detecting temperature changes and biochemical markers, and offer potential avenues for enhancing wound care while preserving the embodied and adaptive nature of nursing work [75]. However, those technologies primarily rely on single biophysical markers or visual analysis, and struggle to capture the multi-sensory dimensions of wound care that nurses consider essential. Furthermore, the acceptance of these systems is often obstructed by a lack of clarity and explainability, especially when black box algorithms are employed. Previous studies have demonstrated that while AI can classify wound types based on photographs, it lacks the ability to interpret tactile sensations, detect odors, or assess the dynamic progression of healing [60]. This highlights a fundamental gap between technological standardization and the context-sensitive, embodied expertise required in wound care. Additionally, studies on AI integration in healthcare have pointed to the risk of over-reliance on algorithmic recommendations, which can lead to the deskilling of healthcare professionals and the marginalization of embodied expertise in clinical decision-making [69, 76].

### Adaptation in home healthcare: opportunities and challenges for AI

The improvisational nature of nursing work in home healthcare emerged as another key finding. Nurses highlighted the intricate interplay of health factors that encompasses broader health information beyond the wound itself. As acknowledged in previous research, the nurses were not focusing on the ‘hole in the patient’ but rather the whole patient [77]. This adaptability allowed them to meet the complex, individualized needs of patients in dynamic home environments. While our findings align with research on the adaptability of healthcare professionals in resource-constrained settings [78], this study also extends these insights by illustrating the specific challenges posed by rigid, standardized AI technologies in such dynamic care contexts. AI technologies may struggle to accommodate the variability and complexity of real-world care [79, 80]. However, AI can fuse data from imaging, laboratory tests, and patient histories to provide more comprehensive analysis and decision-making [81], but it still remains a problem that healthcare data are often incomplete and inconsistent [82]. Insights from previous research [83–85] similarly highlight the challenges of standardizing care practices through AI, emphasizing that technologies must be designed with flexibility to accommodate the variability of nursing, especially in home healthcare.

### Theoretical and practical implications

The findings of this study have significant theoretical and practical implications. Theoretically, they contribute to debates about the role of technology in healthcare by demonstrating that AI should align with the relational, embodied, and adaptive dimensions of care. This challenges reductionist perspectives that focus solely on technical knowledge or readiness, and calls for a broader understanding of technology integration that accounts for the complexities of nursing work.

Mol’s approach foregrounds the daily practices of nurses. By not studying the effectiveness of different wound treatments, but instead including how nurses approach different situations in wound care depending on their judgment of the situation, it is possible to discover the complexity of wound care practices. For example, our study shows that the nurses take bodily parameters into account but seek to understand the patient as a subject, too. They imagine what a situation feels like or means to the patient, mindful that the patient is living with a condition and is experiencing bodily illness, agony, or pleasure.

### Limitations and future research

This study explored municipal home healthcare nurses’ perspectives on wound care and the opportunities and challenges with the integration of AI technologies into those practices. By focusing on nurses’ general perceptions of AI, the study captured broad reflections on its potential impact across various care contexts. This allowed nurses to articulate overarching concerns, expectations, and professional values regarding AI in wound care, rather than being constrained by a single technology or implementation. However, this broad perspective also presents a limitation: as most participants had limited or no direct experience with AI applications in their clinical practice, their reflections were often based on anticipated rather than lived experiences. This introduces a degree of abstraction and may limit the specificity of the findings. Future research could address this limitation by examining the implementation and actual use of specific AI technologies, thereby generating more practice-based insights into how such tools influence nursing work, professional judgment, and patient care. Additionally, while our study focused on nurses’ perspectives, future research could explore the perspectives of AI developers and designers, examining how their assumptions, technical priorities, and decision-making processes align with or diverge from nurses’ needs and care practices. Investigating how nurses and developers interact during AI implementation could provide valuable insights into whether current design processes sufficiently accommodate the relational and embodied aspects of nursing.

Furthermore, while the study was conducted in municipal home healthcare in Sweden, its themes resonate with broader trends in nursing and AI integration. Future research could examine similar issues in other healthcare settings or geographic regions, particularly in hospitals, long-term care facilities, or different national healthcare systems, to explore how contextual factors may shape the integration of AI and digital technologies. Comparative studies could help identify best practices for AI adoption and address structural barriers that influence its implementation in diverse care environments.

## Conclusions

This study explored home healthcare nurses’ perceptions of wound care and the opportunities and challenges posed by AI integration into wound care practices. The findings show that nurses perceive wound care in home healthcare as fundamentally shaped by relational, embodied, and adaptive practices. Trust-building, sensory judgment, and situational responsiveness were described as central to delivering quality care in patients’ homes.

Nurses identified several opportunities for AI to support their work, particularly in relation to documentation, wound image analysis, and communication within the care team. These technologies were seen as potentially helpful when they could reduce administrative burden or enhance decision-making without disrupting the human dimensions of care. At the same time, nurses expressed concerns about the challenges of integrating AI into wound care. Specifically, they questioned whether AI systems can capture the sensory, relational, and context-sensitive aspects of their practice. There were fears that increased standardization might constrain the flexibility and clinical judgment required in home settings.

Finally, by interpreting the findings through Mol’s (2008) concept of the logic of care, the study offers deeper insight into nurses’ perceptions, particularly their concerns about AI integration. Mol’s framework emphasizes that good care is enacted through situated responsiveness, not predefined protocols. This perspective helped illuminate how nurses’ judgments are shaped by the dynamic interplay between patient needs, relational work, and environmental uncertainty. In doing so, the study contributes to broader discussions on technology in healthcare by showing that successful AI integration must go beyond technical efficiency and align with the values, reasoning, and adaptive practices that define professional nursing care. These insights reinforce the need for AI development that is attuned to the lived realities of nursing work. Rather than focusing solely on automation or efficiency, future technologies should be designed to complement the relational, embodied, and adaptive dimensions of care. Further research could explore how AI integration affects professional nursing values, autonomy, and the quality of care in real-world contexts.

## Electronic supplementary material

Below is the link to the electronic supplementary material.

Supplementary Material 1

Supplementary Material 2

## Data Availability

The data and materials of the current study are not publicly available due to confidentiality reasons but are available from the corresponding author on reasonable request.
