# Supplementary material for: Navigating artificial intelligence in home healthcare: challenges and opportunities in nursing wound care
Source: BMC Nurs. 2025 Jun 19;24:660. doi: 10.1186/s12912-025-03348-7 (PMC12180238; doi:10.1186/s12912-025-03348-7)
Supplement: Supplementary file 1 — Supplementary Material 1 [file 12912_2025_3348_MOESM1_ESM.pdf]

COREQ (COnsolidated criteria for REporting Qualitative research) Checklist

Title: Navigating Artificial Intelligence in Home Healthcare: Challenges and Opportunities in Nursing Wound Care

**Topic**	**Item No.**	**Guide Questions/Description**		**Reported on Page No.**
**Domain 1: Research team and reflexivity**				
Personal characteristics				
Interviewer/facilitator	1	Which author/s conducted the interview or focus group?<br><br>Interviews were conducted by SK (n=11), and DT (n=3).		Page 5
Credentials	2	What were the researcher's credentials? E.g. PhD, MD<br><br>**Sara Karnehed** (SK) MSc in Nursing<br>**Ingrid Larsson** (IL) PhD<br>**Lena Petersson** (LP) PhD<br>**Lena-Karin Erlandsson** (LKE) PhD<br>**Daniel Tyskbo** (DT) PhD		
Occupation	3	What was their occupation at the time of the study?<br><br>**Sara Karnehed** (SK) MSc in Nursing, PhD student at Halmstad University (School of Health and Welfare). sara.karnehed@hh.se.<br><br>**Ingrid Larsson**, (IL) Registered Nurse, PhD, Professor at Halmstad University (School of Health and Welfare). ingrid.larsson@hh.se<br><br>**Lena Petersson**, (LP) PhD, Senior Lecturer at Halmstad University (School of Health and Welfare). lena.petersson@hh.se<br><br>**Lena-Karin Erlandsson**, (LKE) PhD, Professor at Halmstad University (School of Health and Welfare), Dean (School of Health and Welfare). lena-karin.erlandsson@hh.se<br><br>**Daniel Tyskbo**, (DT) PhD, Assistant Professor at Halmstad University (School of Health and Welfare). daniel.tyskbo@hh.se		
Gender	4	Was the researcher male or female?<br><br>Daniel Tyskbo (DT) is male, Ingrid Larsson (IL), Lena-Karin Erlandsson (LKE), Lena Petersson (LP) and Sara Karnehed (SK) are female.		
Experience and training	5	What experience or training did the researcher have?<br><br>Sara Karnehed ORCID: 0000-0002-5257-2524. Her PhD project focuses on the use and implementation of information-driven care in home		

		healthcare with a special focus on how it affects nurses' work environment and profession.<br><br>Ingrid Larsson, ORCID: 0000-0002-4341-660X. Her research is related to person-centered care and the implementation of AI in health care.<br><br>Lena Petersson ORCID: 0000-0001-7874-7970. Her research is related to how knowledge and information practices change in a digital society, information transparency, and digitalization in the healthcare sector.<br><br>Lena-Karin Erlandsson ORCID: 0000-0001-5865-2632. Her research focuses on people's everyday lives and its impact on health.<br><br>Daniel Tyskbo ORCID: 0000-0002-3727-6153. His research is within the field of information-driven care with a special focus on the implementation process of AI in the healthcare context.		
Relationship with participants				
Relationship established	6	Was a relationship established prior to study commencement?<br><br>No relationship was established prior to the study.		Page 5-6
Participant knowledge of the interviewer	7	What did the participants know about the researcher? e.g. personal goals, reasons for doing the research.<br><br>Both oral and written informed consent were obtained from all participants, ensuring they were fully aware of the study's purpose, procedures, potential risks, and their rights to withdraw at any time prior to participation.		Page 7
Interviewer characteristics	8	What characteristics were reported about the inter viewer/facilitator? e.g. Bias, assumptions, reasons and interests in the research topic.<br><br>The interviews were held in Swedish by an independent, experienced researcher (DT) (n=3) and by a supervised researcher experienced in home healthcare nursing and interviewing (SK) (n=11).		Page 5
**Domain 2: Study design**				
Theoretical framework				
Methodological orientationand Theory	9	What methodological orientation was stated to underpin the study? e.g.grounded theory, discourse analysis, ethnography, phenomenology, content analysis.<br><br>The research method was inspired by the method outlined by Gioia (Gioia, 2020; Gioia et al., 2013).		Page 6
Participant selection				

Sampling	10	How were participants selected? e.g. purposive, convenience, consecutive, snowball.<br><br>A purposeful sampling approach was employed to recruit nurses in the two municipalities. Participants were recruited based on the following inclusion criteria: (1) registered nurses working in the municipality's home healthcare organization and (2) experience in wound care within home healthcare settings. The researchers contacted the managers of each home healthcare organization and asked for assistance in recruiting nurses.		Page 5
Method of approach	11	How were participants approached? e.g. face-to-face, telephone, mail, email.<br><br>Participants were posted a mail of invitation and information sheet.		Page 5
Sample size	12	How many participants were in the study?<br><br>The sample consisted of 14 nurses.		Page 5
Non-participation	13	How many people refused to participate or dropped out? Reasons?<br><br>Nurses who declined to participate attributed their decision to competing commitments, such as work obligations.		Page 5
Setting				
Setting of data collection	14	Where was the data collected? e.g. home, clinic, workplace.<br><br>Interviews were completed, with sessions held either on-site at the workplaces of nurses (n=5) or via video call where necessary due to logistical considerations (n=9).		Page 5
Presence of non-participants	15	Was anyone else present besides the participants and researchers?<br><br>No one else was present besides the participant and researcher during the interviews.		Page 5
Description of sample	16	What are the important characteristics of the sample?<br><br>Nurses were recruited in two municipalities situated on the Swedish West Coast. Each municipality covers an area of around 1,000 square kilometers and has a population density of 11 people per square kilometer and 28 people per square kilometer, respectively. The participants had individual experience in wound management ranging from 1 to 27 years. They engaged in wound care activities 1 to 5 days per week.		Page 5
Data collection				
Interview guide	17	Were questions, prompts, guides provided by the authors? Was it pilot tested?<br><br>The interviews were conducted using a protocol		Page 6

		that included semi-structured, open-ended questions, addressing perceptions of wound care practices and AI (see additional file 2). The protocol questions were developed for this study. Follow-up questions were asked when further exploration and clarification was needed. The questions were not pilot-tested.		
Repeat interviews	18	Were repeat interviews carried out? If yes, how many?<br><br>No repeat interviews were carried out.		
Audio/visual recording	19	Did the research use audio or visual recording to collect the data?<br><br>Interviews were audio recorded.		Page 6
Field notes	20	Were field notes made during and/or after the interview or focus group?<br><br>No field notes were made during or after the interviews.		
Duration	21	What was the duration of the interviews or focus groups?<br><br>The duration of the interviews was approximately 60 minutes.		Page 6
Data saturation	22	Was data saturation discussed?<br><br>Data saturation was not discussed.		
Transcripts returned	23	Were transcripts returned to participants for comment and/or correction?<br><br>Transcripts were not returned to participants.		
**Domain 3: analysis and findings**				
Data analysis				
Number of data coders	24	How many data coders coded the data?<br><br>Two researchers (SK and DT) coded the data.		Page 6
Description of the coding tree	25	Did authors provide a description of the coding tree?<br><br>No coding frame was provided.		
Derivation of themes	26	Were themes identified in advance or derived from the data?<br>Themes derived from the data.		Page 6-7
Software	27	What software, if applicable, was used to manage the data?<br>No. Not applicable.		
Participant checking	28	Did participants provide feedback on the findings?<br><br>No. Participants were not provided feedback on the findings.		
Reporting				
Quotations presented	29	Were participant quotations presented to illustrate the themes/findings? Was each quotation identified? e.g. participant number.		Page 8-15

		Quotations have been presented throughout the result section with participant codes assigned to all participants and used against quotations.		
Data and findings consistent	30	Was there consistency between the data presented and the findings?<br><br>Yes. There was consistency between the data presented and the findings.		
Clarity of major themes	31	Were major themes clearly presented in the findings?<br><br>Yes. Major themes were clearly presented in the findings.		Page 8-15
Clarity of minor themes	32	Is there a description of diverse cases or discussion of minor themes?<br><br>Yes. Minor themes were clearly presented in the findings.		Page 8-15

Tong A, Sainsbury P, Craig J. Consolidated criteria for reporting qualitative research (COREQ): a 32-item checklist for interviews and focus groups. *International Journal for Quality in Health Care*. 2007. Volume 19, Number 6 pp. 349 - 357
