# Supplementary material for: Navigating artificial intelligence in home healthcare: challenges and opportunities in nursing wound care
Source: BMC Nurs. 2025 Jun 19;24:660. doi: 10.1186/s12912-025-03348-7 (PMC12180238; doi:10.1186/s12912-025-03348-7)
Supplement: Supplementary file 2 — Supplementary Material 2 [file 12912_2025_3348_MOESM2_ESM.pdf]

Additional file 2. Interview guide

- Can you describe your work and your working days to me?
- Do you use AI systems and other digital systems in your workplace?
- How do you experience the use of AI and other digital systems in your work?

- Can you tell me about your experiences of providing wound care?
- Can you describe what the wound care process for patients looks like today?
- Can you tell me how you perform wound care?
- What is important to achieve good wound care?
- What challenges do you experience in your work with wound care?

- What do you think about the possibilities of use of AI in wound care?
- Where in the care processes, and how, could AI help you in your work with wound care?
- Do you have any thoughts on how an AI application in wound care would affect your care work?
- Do you have any thoughts on how an AI application in wound care would affect patients and wound care outcomes?
- Is there anything else that you think is important that concerns wound care and AI that we haven't talked about?
